# Preparation of Chlorophyll Nanoemulsion from Pomelo Leaves and Its Inhibition Effect on Melanoma Cells A375

**DOI:** 10.3390/plants10081664

**Published:** 2021-08-12

**Authors:** Man-Hai Liu, Yi-Fen Li, Bing-Huei Chen

**Affiliations:** 1 Department of Food Science, China University of Science and Technology, Taipei 11581, Taiwan; manhailiu@gmail.com; 2 Department of Food Science, Fu Jen Catholic University, New Taipei City 24205, Taiwan; kim06060515@gmail.com; 3 Department of Nutrition, China Medical University, Taichung 404, Taiwan

**Keywords:** pomelo leave, chlorophyll, nanoemulsion, human melanoma cells A375

## Abstract

Pomelo (*Citrus grandis*), an important fruit crop grown in tropical and subtropical areas, is cultivated mainly in Asian countries. The dominant pigment in pomelo leaves, chlorophyll, has been reported to possess many biological activities such as antioxidant, anti-inflammation and anticancer. The objectives of this study were to determine chlorophylls in Pomelo leaves by high-performance liquid chromatography-mass spectrometry (HPLC-MS) and to encapsulate the isolated chlorophylls from preparative column chromatography into a nanoemulsion system for elucidating the inhibition mechanism on the growth of melanoma cells A375. The results showed that chlorophyll a and chlorophyll b could be separated within 25 min by using a C18 column and a gradient ternary mobile phase of acetone, acetonitrile and methanol. Pomelo leaves mainly contained chlorophyll a (2278.3 μg/g) and chlorophyll b (785.8 μg/g). A highly stable chlorophyll nanoemulsion was prepared with the mean particle size being 13.2 nm as determined by a dynamic light scattering (DLS) method. The encapsulation efficiency of chlorophyll nanoemulsion was 99%, while the zeta potential was −64.4 mV. In addition, the chlorophyll nanoemulsion possessed high thermal stability up to 100 °C and remained stable over a 90-day storage period at 4 °C. Western blot analysis revealed that chlorophyll nanoemulsion and extract could upregulate p53, p21, cyclin B and cyclin A as well as downregulate CDK1 and CDK2 in a concentration-dependent manner for inhibition of melanoma cells A375. Furthermore, chlorophyll nanoemulsion and extract could upregulate Bax and cytochrome C and downregulate Bcl-2, leading to activation of caspase-9, caspase-8 and caspase-3 for the induction of cell apoptosis. Compared to chlorophyll extract, chlorophyll nanoemulsion was more effective in inhibiting the growth of melanoma cells A375.

## 1. Introduction

Pomelo (*Citrus grandis* (Linn.) Osbeck), a vital plant possessing biological activities, is grown in more than 50 countries/regions, predominantly in China, southern Japan, Thailand, Vietnam, Indonesia, and the United States [1]. Studies have shown that pomelo leaves, a waste often discarded, also contained high level of chlorophylls, the most widely distributed photosynthetic pigment in nature, especially in green plants and algae [2]. Of the various chlorophylls, many research groups have investigated the biological activities of chlorophyllin, a water-soluble derivative of chlorophylls shown to exert potent antimutagenic activity in prokaryotic and eukaryotic organisms in vitro [3,4] Additionally, several in vivo experiments have revealed that chlorophyllin derived from rainbow trout could minimize DNA damage and liver cancer induced by aflatoxin B1 [5,6].

The association between cell apoptosis and cancer, as well as various autoimmune diseases, has been well documented. Accordingly, the apoptotic pathways can be divided into intrinsic and extrinsic pathways, with the former mediated by the mitochondria and the latter by death receptors, while both pathways can lead to apoptosis via caspase activation. More specifically, the intrinsic pathway can be initiated by tumor suppressors such as p53, a transcription factor activated by DNA damage, through stimulation of the expression of Bcl-2 family members such as Bax and Bad, resulting in a release of substances that reside between the inner and outer mitochondrial membranes such as cytochrome C and Smac/DIABLO. In addition, the so-called apoptotic body, composed of cytochrome C, Apaf-1 and procaspase 9 in cytosol, is the activated form of caspase-9. In the presence of caspase-8, caspase-3 can be activated by caspase-9 for apoptosis execution [7]. For the extrinsic pathway, the death receptors Fas (CD95), tumor necrosis factor receptor (TNFR1 and TNFR2) and death ligands (FasL, TNF) on the cell membrane are activated when stimulated by the death signal, followed by binding of the death ligands to death receptors for formation of Fas-associated protein with death domain (FADD), leading to caspase-8 activation and formation of a death-inducing signaling complex (DISC). Finally, caspase-3 is activated for apoptosis execution [8]. Many studies have demonstrated that chlorophylls and their derivatives can induce cancer cell apoptosis. For instance, sodium copper chlorophyllin (SCC) was shown to activate caspase-8/caspase-3/poly (-ADP-ribose) polymerase for nuclear condensation, thereby promoting apoptosis of human colon cancer cells (HCT116) [9]. In another study, Chan et al. [10] reported that the water-soluble pheophorbide a could induce apoptosis in human hepatoma cells (Hep3B) via the cytochrome C–mediated apoptotic pathway, followed by activation of caspase-3/caspase-9. In addition, SCC was shown to induce apoptosis in cancer cells through inhibition of the oncoprotein mitogen-activated protein kinase (MAPK) and signal transduction by the extracellular signal-regulated kinase (ERK) in breast cancer cells MCF-7 [11]. Wu et al. [12] further illustrated that chlorophyll a in *Ludwigia octovalvis* could induce apoptosis of 3T3-L1 adipocytes through activation of the CD95 system for regulation of Bcl-2 family protein and activation of caspase-3, leading to the activation of AMP activated protein kinase (AMPK) signaling pathway for the inhibition of adipogenesis. In addition, both chlorophyll and chlorophyllin were shown to exhibit antimutagenic and anticancer properties in vivo and in vitro, probably caused by the binding of both to chemical mutagens or their metabolites for toxicity reduction [13]. All of these findings indicated that chlorophylls and their derivatives possessed a great potential for their development into an anticancer drug. As pomelo leaves are often discarded as waste after harvest, it will be a great advantage to the food or drug industry if the chlorophylls in pomelo leaves can be extracted and developed into a functional food or anticancer drug.

In recent years nanotechnology has been widely used in many areas such as energy, materials, cosmetics, foods, drugs and medicine. In particular, there has been a great upsurge in the development of nanodelivery systems for innovative therapy through active or passive targeting of plant extract-containing bioactives for enhanced solubility and bioavailability. For instance, Vassallo et al. [14] reported that the aqueous extract of polyphenol-rich *Hura crepitans*, encapsulated into a nanoliposome system with a particle size of 84 nm, polydispersity index of 0.2 and zeta potential of −46 mV, could effectively reduce the production of reactive oxygen species (ROS) in HepG2 cells at a concentration of 3.125 μg/mL. Likewise, a similar reduction in ROS and an enhancement of antioxidant activity were also shown for ethanolic *Capsicum annuum* extract after encapsulation into a nanoliposome system (particle size 83.8 nm) through promoting the expression of endogenous antioxidants such as catalase, superoxide dismutase and glutathione peroxidase in a nuclear erythroid 2-related factor 2 (Nrf-2) pathway [15]. Of the various nanoformulation techniques, nanoemulsion is widely used for product development in the food and pharmaceutical industries to generate droplets in O/W or W/O emulsion systems with a particle size ranging from 10–100 nm. Most importantly, nanoemulsion is known to possess the ability to encapsulate unstable bioactive compounds, thereby enhancing aqueous solubility, heat and storage stability, as well as in vivo bioavailability [16]. Due to the unstable nature of chlorophylls under heating and acidic conditions, it is necessary to use an appropriate encapsulation technique such as nanoemulsion to improve chlorophyll stability and biological activity. To the best of our knowledge, this is the first paper dealing with the inhibition of cancer cells by chlorophyll nanoemulsion. The objectives of this study were to isolate chlorophylls from pomelo leaves for nanoemulsion preparation and study their inhibition effects on human melanoma cells A375.

## 2. Results and Discussion

### 2.1. HPLC Analysis of Chlorophylls in Pomelo Leaves

**Figure 1:**
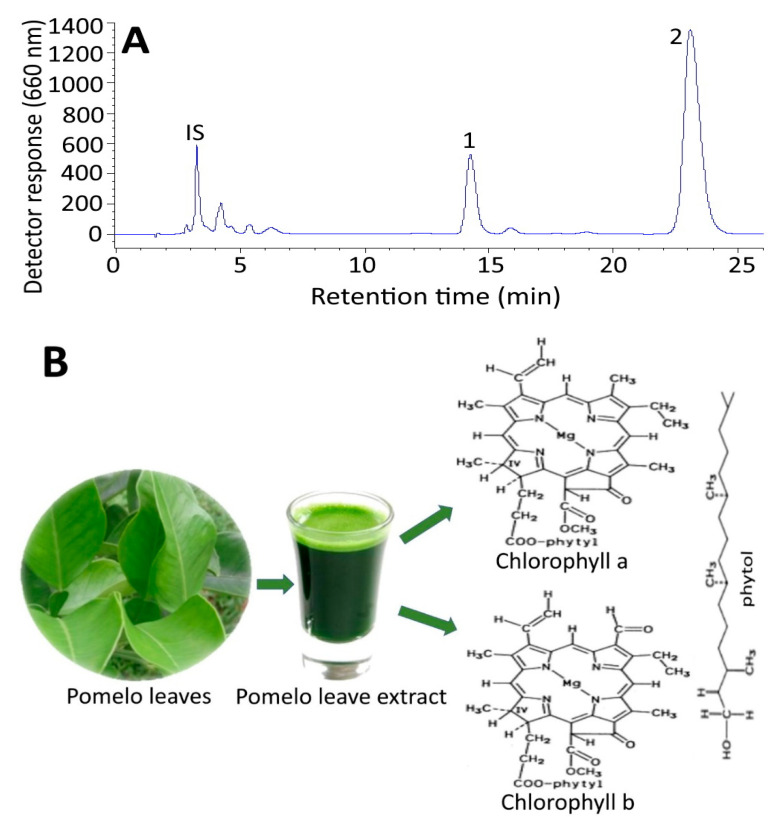
HPLC chromatogram of chlorophylls in pomelo extract (**A**) Peaks: IS, internal standard Fast Green FCF; 1, chlorophyll b; 2, chlorophyll a and (**B**) a schematic diagram showing the chemical structures of isolated chlorophyll a and chlorophyll b compounds from pomelo leaves.

Figure 1 shows the HPLC chromatogram of chlorophylls in pomelo leave extract. An adequate separation of chlorophyll b and chlorophyll a was attained with the retention time being 13.92 and 22.54 min and the retention factor of 3.35 and 6.04, respectively (Table 1). This outcome implied that the optimal solvent strength of the mobile phase was controlled. Table 1 also shows the mass to charge ratio (*m*/*z*) and the maximum absorption wavelength (λ_max_) in pomelo leaves. Based on the identification criteria described in the method section [17], both chlorophylls a and b were present in pomelo leaves and the contents were determined to be 2278.3 μg/g and 785.8 μg/g, respectively. The regression equations used for quantitation of chlorophylls a and b were y = 16.642x + 0.1786 (R^2^ = 0.9999) for the former and y = 9.095x−0.2035 (R^2^ = 0.9999) for the latter.

### 2.2. Chlorophyll Nanoemulsion Characteristics

**Figure 2:**
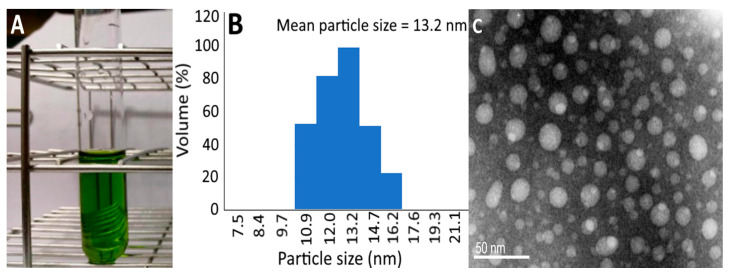
The appearance of chlorophyll nanoemulsion (**A**) and its particle size distribution as determined by a dynamic light scattering instrument (**B**) as well as the transmission electron micrograph imaged at 120 kVa (**C**).

A clear and transparent chlorophyll nanoemulsion is shown in Figure 2a, which may be due to the presence of nanoparticles with size < 100 nm without agglomeration. Additionally, the particle size distribution, as determined by DLS, revealed a mean particle size of 13.2 nm (Figure 2b) and the TEM micrograph depicted the shape of the chlorophyll nanoemulsion to be roughly round in shape (Figure 2c). Only a slight change in zeta potential, particle size, PDI and pH was shown over a 3-month storage period at 4 °C (Table 2), implying a high stability of this nanoemulsion prepared in our study. Similarly, only a minor change in particle size was observed during heating at 40–100 °C for 2 h (Table 3). However, the zeta potential decreased to −28.6 and −25.8 mV after heating at 80 and 100 °C for 2 h, respectively, indicating that the heating temperature and time length should be controlled at 100 °C (≤1.5 h) to maintain zeta potential at ≤−30 mV for the enhancement of chlorophyll nanoemulsion stability [16].

### 2.3. Cell Tolerance to Sample Solvent and Blank Nanoemulsion

**Figure 3:**
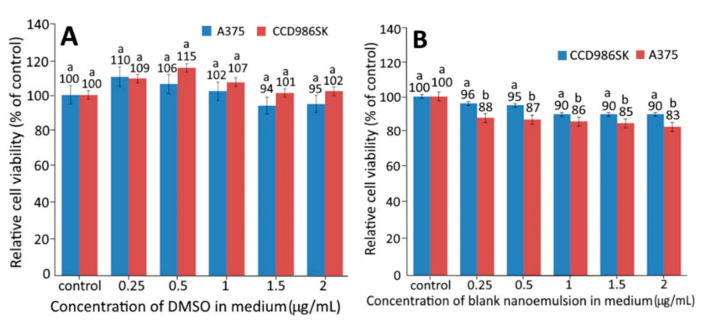
Effect of different concentrations of DMSO (**A**) and blank nanoemulsion (**B**) on growth of fibroblast cells CCD966SK and human melanoma cells A375 after 48 h incubation as measured by MTT assay. Data represent mean ± standard deviation (*n* = 3) with different small letters (a,b) indicating significantly different values at *p* < 0.05.

In this experiment, DMSO was used as a solvent, while lecithin and Tween 80 were used for nanoemulsion preparation. Therefore, to avoid the interference of DMSO and nanoemulsion components with cell growth, we needed to evaluate the tolerance of human malignant melanoma cells (A375) and fibroblast cells (CCD-986SK) to DMSO and blank nanoemulsion, and the results are shown in Figure 3A,B. With DMSO concentration at between 0.25 and 2.00%, the viability of both CCD986SK and A375 cells exceeded 94%, which was not significantly different from that of the control (*p* > 0.05) (Figure 3A). Similarly, when the blank nanoemulsion concentration was 2%, the viability of CCD986SK and A375 cells was 90 and 83%, respectively, and the difference was significant (*p* < 0.05). A similar result was observed for the blank nanoemulsion concentration from 0.25 to 1.5%. Thus, to maintain high viability of both CCD986SK and A375 cells, the dose of both DMSO and blank nanoemulsion was controlled at 1% for subsequent experiments.

In several previous studies Arechabala et al. [18] compared the effects of six surfactants on the viability of human fibroblast cells (CCD986SK) and reported that cell viability decreased in the following order: Tween 80 > Texapon N40 > Tween 60 > Texapon K1298 > Triton X−100 > benzethonium chloride, indicating that Tween 80 possessed the lowest cytotoxicity towards CCD986SK cells. In a later study, Li et al. [19] studied the effect of different concentrations of Tween 20 on colon cancer cells HT29 and found that a high viability was maintained when the dose was <0.02%. In addition, the viability of colon cancer cells Caco2/TC7 was shown to be insignificantly different (*p* > 0.05) compared with control when the DMSO dose was between 2 and 10% [20]. However, the viability dropped to 73% when the DMSO dose reached 20%. Apparently, the cell viability can be varied depending on the amount and variety of solvents and surfactants used.

### 2.4. Cell Growth Inhibition Assay

**Figure 4:**
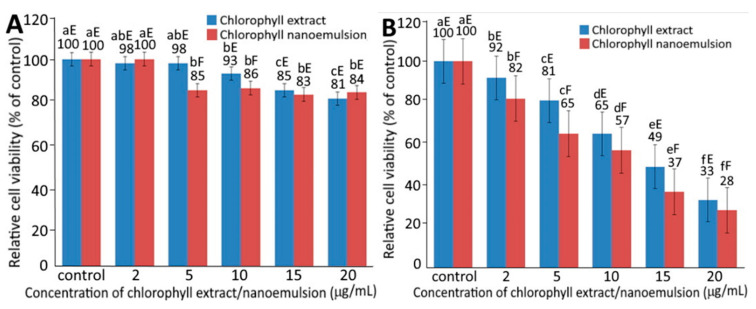
Effect of different concentrations of chlorophyll extract and nanoemulsion on growth of fibroblast cells CCD986SK (**A**) and human melanoma cells A375 (**B**). For control, cells were incubated in medium containing 1% DMSO. Data represent mean ± standard deviation (*n* = 3) with different small letters (a–f) within the same treatment for different concentrations as well as different capital letters (E,F) within the same concentration for different treatments indicating significantly different values at *p* < 0.05.

The effect of different concentrations of chlorophyll extract (A) and chlorophyll nanoemulsion (B) on the growth of fibroblast cells CCD-986SK and melanoma cells A375 is shown in Figure 4A,B, respectively. A dose-dependent decrease in the viability of CCD-986SK and A375 cells was shown for both chlorophyll extract and nanoemulsion. More specifically, following the treatment of chlorophyll extract and nanoemulsion at 2, 5, 10, 15 and 20 μg/mL, the viability of CCD-986SK cells was respectively 98, 98, 93, 85 and 81% for the former, and 100, 85, 86, 83 and 84% for the latter. Comparatively, chlorophyll extract possessed less toxicity towards CCD-986SK cells than chlorophyll nanoemulsion. Nevertheless, both chlorophyll extract and nanoemulsion exhibited a slight inhibition effect on CCD-986SK cells, suggesting a mild toxicity towards fibroblast cells.

For melanoma cells A375, the viability decreased respectively to 92, 81, 65, 49 and 33% for chlorophyll extract as well as 82, 65, 57, 37 and 28% for chlorophyll nanoemulsion at doses of 2, 5, 10, 15 and 20 μg/mL. By comparison, chlorophyll nanoemulsion possessed a higher toxicity on A375 cells than chlorophyll extract, as evident by an IC_50_ of 3.3 and 15.6 μg/mL, respectively. It can be postulated that, compared to chlorophyll extract, chlorophyll nanoemulsion should be more efficient in its diffusion from the extracellular matrix into the cytoplasm and nucleus for antitumor efficiency through an enhanced permeability and retention (EPR) effect for passive targeting [21].

In a study dealing with the treatment of human cholangiocarcinoma cells (QBC-939) with chlorophyll derivatives (HEPa) for 24 h, the viability of QBC-939 cells was not significantly reduced (*p* > 0.05) when the HEPa dose was <8 µM. However, when the HEPa dose exceeded 8 µM, the cell viability reduced slightly to 84.53%. Additionally, the viability of QBC-939 cells was 27.52 and 7.16% respectively, when exposed to a light intensity of 2 and 8 J/cm^2^ in the presence of 8 µM HEPa, implying that a higher light intensity could greatly reduce the viability of QBC-939 cells [22]. Another chlorophyll derivative, bacteriochlorophyll-serine, a photosensitizer that can be activated by near-infrared light at 780 nm for deep penetration into body tissues and possible application to the treatment of large and solid cancers, was shown to destroy cancer cells and be effective at low oxygen concentration. Furthermore, the activity of chlorophyll derivatives can be attributed to their ability in modulating mutagen/carcinogen bioavailability, metabolism and, ultimately, decreasing the exposure of humans at risk to carcinogens [23].

### 2.5. Cell Cycle Analysis

There was no significant difference (*p* > 0.05) in sub-G1, G0/G1 and S phases between the control and the other two chlorophyll concentrations (5 and 10 μg/mL) (Figure S1). However, the proportion of G2/M phase followed a dose-dependent increase and reached 28.5% following treatment with chlorophyll nanoemulsion at 10 μg/mL, which was substantially higher than the control treatment (17.1%). The same trend was also observed for chlorophyll extract. Thus, it can be concluded that the growth of melanoma cells A375 was arrested at the G2/M phase following treatment with chlorophyll extract or nanoemulsion prepared from pomelo leaves. In a similar study, Yang, Hung and Chen [16] also reported that the cell cycle of melanoma cells B16-F10 was arrested at the G2/M phase following treatment with coffee oil–algae oil nanoemulsion. However, in other studies Albino et al. [24] (2000) reported that the cell cycle of melanoma cells SK-Mel-29 and SK-Mel-110 was arrested at G0/G1 and S phases following treatment with DHA. Siddiqui et al. [25] and Merendino et al. [26] studied the effect of DHA on the cell cycle of leukemia cell clone-E6-1 and pancreatic cancer cell PaCa-44, respectively, and the cell cycle was arrested at S phase for the former and G0/G1 phase for the latter. Apparently, the cell cycle phase distribution and arrest can be dependent upon the cancer cell type, dose and composition of nanoemulsion.

Accordingly, cell growth needs to pass through the four phases of G1, S, G2 and M, and the cell cycle plays an important role in the screening of anticancer drugs. The process of cell replication and division requires cyclins, cyclin-dependent kinases, regulatory subunits (cyclin A, B, D or E) and some inhibitors of cyclin kinases (CKIs). When cell cycle hormones are overexpressed in the human body, they often cause the production of cancer cells, such as the overexpression of cyclins and CDKs or the inhibition of CDK inactivation. For example, in human cancers, it can be found that the regulation of cyclin D1-Rb is abnormal, while in human malignant tumors, the accumulation of cyclin D1 can be shown.

Based on the result of MTT assay, both chlorophyll extract and nanoemulsion, at a dose of 5, 10 and 20 μg/mL, were selected for studying the expression of the cell cycle- and apoptosis-related proteins. Thus, the expression of proteins associated with the regulation of the cell cycle, such as cyclin A, cyclin B, CDK1 and CDK2, as well as CDK inhibitors such as p53 and p21, and the expression of proteins associated with apoptosis, such as Bcl-2, Bax and cytochrome C, need to be investigated.

### 2.6. Expression of Cell Cycle- and Apoptosis-Related Proteins

**Figure 5:**
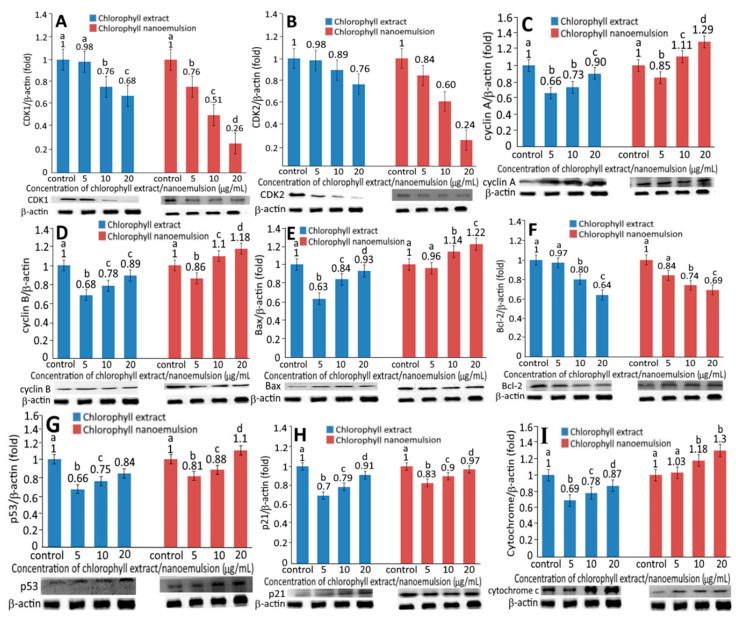
Effect of chlorophyll extract and nanoemulsion on protein expressions of CDK1 (**A**), CDK2 (**B**), cyclin A (**C**), cyclin B (**D**), Bax (**E**), Bcl-2 (**F**), p53 (**G**), p21 (**H**) and cytochrome C (**I**). For control, cells were incubated with medium only. Data represent mean ± standard deviation (*n* = 3) with different letters (a–d) within the same treatment indicating significantly different values at *p* < 0.05.

Following the treatment of chlorophyll extract or nanoemulsion at 5, 10 and 20 μg/mL for 48 h, the protein expression of CDK1 and CDK2 in human melanoma A375 cells relative to that in the control group are shown in Figure 5A,B, respectively. A dose-dependent decrease in both CDK1 and CDK2 expressions was shown for both chlorophyll extract and nanoemulsion. By comparison, chlorophyll nanoemulsion was more effective in reducing CDK1 expression than chlorophyll extract at the same dose. The same trend was also observed for CDK2. However, by comparing both treatments at 5, 10 or 20 μg/mL, the expression level of CDK1 was reduced to a higher extent than CDK2. In a recent study, Mughees and Wajid [27] pointed out that the encapsulation of herbal extracts by polymeric nanoparticles is a prominent and effective way of targeted drug delivery for cancer. Furthermore, it has been well documented that the bioactive compounds encapsulated in nanoemulsion can enhance their solubility, controlled release, absorption in the gastrointestinal tract and penetration into cells [16,21] Thus, the nanoemulsion prepared in our study should be able to diffuse into melanoma cells to reduce both CDK1 and CDK2 expressions with arrest at G2/M phase. Figure 5C,D show the expression levels of cyclin A and cyclin B as affected by chlorophyll extract and nanoemulsion. A dose-dependent increase in cyclin A and cyclin B expressions was shown for both chlorophyll extract and nanoemulsion. However, chlorophyll nanoemulsion was more effective in enhancing cyclin A and cyclin B expressions than chlorophyll extract at the same dose. Comparatively, the difference between cyclin A and cyclin B expressions was minor following the treatment of chlorophyll extract or nanoemulsion at 5, 10 and 20 μg/mL. Figure 5G,H shows the effect of chlorophyll extract and nanoemulsion on p53 and p21 expression in human melanoma cells. A dose-dependent rise in p53 and p21 expression was shown for both chlorophyll extract and nanoemulsion. However, at the same dose, a much higher p53 expression was found for chlorophyll nanoemulsion than for chlorophyll extract, implying that the former could inhibit the activity of cyclin A-CDK2 complex more effectively than chlorophyll extract, resulting in an arrest of the human melanoma cell cycle at the G2/M phase.

In a recent study, Ren et al. [28] studied the effect of berberine on the proliferation, apoptosis and migration of skin melanoma A375 cells and reported a reduced expression of CDK1 and CDK2 as well as an increased expression of cyclin D1 and cyclin A. Thus, berberine could suppress the growth and migration of human melanoma cells, leading to apoptosis. Moreover, berberine at low dose (20 and 40 μM) resulted in a cell cycle arrest at S and G2/M phases, while a high dose of berberine (60 and 80 μM) led to cell cycle arrest at the G2/M phase. Accordingly, cyclin B can form a complex with CDK1 (cyclin B–CDK1) for mitosis and regulation of the G2/M phase, while cyclin A can be activated at the S and G2 phases for DNA synthesis and mitosis, and formation of cyclin A–CDK2 complex at the S phase, as well as formation of cyclin A–CDK1 complex at the G2 prephase. The above results showed that chlorophyll extract and nanoemulsion could induce the G2/M phase-related protein expression of A375 cells, i.e., the expression of cyclin A and cyclin B was enhanced, while the expression of CDK1 and CDK2 was reduced.

Likewise, a dose-dependent rise in Bax expression was shown for both chlorophyll extract and nanoemulsion (Figure 5E). In addition, chlorophyll nanoemulsion showed a more pronounced effect in elevating Bax expression than chlorophyll extract at the same dose. Bax, a pro-apoptotic protein of the Bcl-2 family, can lower the mitochondrial membrane potential and result in a release of apoptotic signals and related proteins such as Bcl-2 and cytochrome C, leading to the activation of caspase-9 and caspase-3 for subsequent apoptosis [21]. In addition, p53 can not only increase Bax expression through transcriptional regulation but also binds directly to Bcl-2 to promote cell apoptosis [16].

The expression of Bcl-2 in human melanoma A375 cells following treatment with 5, 10 and 20 μg/mL of chlorophyll extract and nanoemulsion for 48 h, relative to the control group, is shown in Figure 5F. Unlike Bax, a dose-dependent decline in Bcl-2 expression was shown for both chlorophyll extract and nanoemulsion. In addition, chlorophyll nanoemulsion was more efficient in decreasing Bcl-2 expression than chlorophyll extract at 5 and 10 μg/mL. However, the difference in Bcl-2 expression between chlorophyll extract and nanoemulsion at 20 μg/mL remained insignificant (*p* > 0.05). As mentioned above, Bcl-2 is an anti-apoptotic protein belonging to the Bcl-2 family, which can maintain the balance between mitochondrial membrane potential and integrity. This prevents cytochrome C release from the mitochondria, thereby inhibiting the activity of the downstream caspase and preventing cells from apoptosis. Collectively, both chlorophyll extract and nanoemulsion could reduce Bcl-2 expression while increasing Bax expression for the induction of apoptosis.

**Figure 6:**
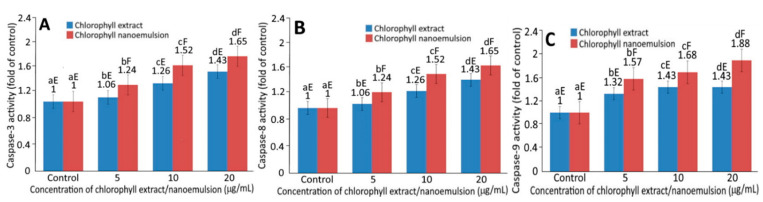
Activities of caspase-3 (**A**), caspase-8 (**B**) and caspase-9 (**C**) in human melanoma cells A375 as affected by different concentrations of chlorophyll extract and nanoemulsion. Data represent mean ± standard deviation (*n* = 3) with different small letters (a–d) within the same treatment for different concentrations as well as different capital letters (E,F) within the same concentration for different treatments indicating significantly different values at *p* < 0.05.

Figure 5G shows the expression level of the tumor suppressor protein p53. A dose-dependent increase in p53 expression was shown for both chlorophyll extract and nanoemulsion. Comparatively, chlorophyll nanoemulsion was more effective in elevating p53 expression than chlorophyll extract at the same dose. Like p53, a dose-dependent rise in p21 expression was shown for both chlorophyll extract and nanoemulsion. Additionally, chlorophyll nanoemulsion was more effective in increasing p21 expression than chlorophyll extract (Figure 5H). In addition, a dose-dependent increase in cytochrome C expression was shown for both chlorophyll extract and nanoemulsion (Figure 5I). Comparatively, chlorophyll nanoemulsion possessed a more pronounced effect in enhancing cytochrome C expression at the same dose. Figure 6 shows the activities of caspase-3, caspase-8 and caspase-9 in human melanoma cells A375 as affected by chlorophyll extract and nanoemulsion at 5, 10 and 20 μg/mL. A dose-dependent increase in the activities of caspase-3, -8 and -9 was shown for both chlorophyll extract and nanoemulsion, and the latter was more efficient than the former in elevating activities of caspase-3, -8 and -9 at the same dose (Figure 6A–C). Furthermore, the caspase-9 activity was raised to a higher level than that of caspase-3 and caspase-8 following the treatment of chlorophyll extract or nanoemulsion at 5, 10 and 20 μg/mL.

Ren, Yang, Li, Yang, Su and Su [28] pointed out that, in addition to inhibiting the activity of the cyclin–CDK complex, p21 could also bind to CDK2 and CDK1 and result in a decrease in CDK protein expression, leading to a rise in cyclin expression for the subsequent arrest of melanoma cells A375 at the G2/M phase. In a similar study, Ma and Zhang [29] treated hepatoma cells HepG2 with diosmetin and observed a rise in the expressions of p21, cyclin A, cyclin B and CDK1, with the cell cycle arrested at the G2/M phase. Yang, Hung and Chen [16] prepared coffee oil–algae oil-based nanoemulsions and studied their effects on the inhibition of melanoma cells B16F10; a dose-dependent increase in cyclin B expression was shown with the cell cycle arrested at the G2/M phase. Yue et al. [30] treated melanoma cells M14 with cardamonin (60~90 μM) and reported that both caspase-8 and caspase-9 activities increased following a rise in incubation time. Yu et al. [31] further reported a rise in caspase-3, caspase-8 and caspase-9 activities following the treatment of melanoma cells M14 with ursodeoxycholic acid, along with a decrease and increase in expressions of Bcl-XL and Bax, respectively, resulting in a release of cytochrome C from the mitochondria into the cytoplasm for subsequent apoptosis. This outcome suggested that the apoptosis pathway is not a single pathway but may include both mitochondria and death receptor pathways. More recently, Do et al. [32] treated human melanoma cells with the aqueous extract from Moringa oleifera leaves; a dose-dependent increase in Bax expression and decrease in Bcl-2 expression was shown, accompanied by a rise in caspase-3 activity for apoptosis execution.

All of these studies shown above have implied that phytochemicals could affect the expression of cell cycle-related proteins in different types of cancer cells, and the expression efficiency could be dependent upon the variety of phytochemicals, type of cancer cells, incubation time and preparation method of phytochemicals. However, in the literature reports there is no information available regarding the effect of chlorophyll nanoemulsion on the inhibition of melanoma cells. In our study, we demonstrated for the first time that both chlorophyll extracts and nanoemulsions led to the apoptosis of melanoma cells A375 through an increase in the expression of cyclin B and cyclin A, and a decrease in the expression of CDK1 and CDK2, as well as elevation of the p53 and p21 expressions for a subsequent rise in Bax expression and decrease in Bcl-2 expression. This, in turn, resulted in an imbalance of the membrane potential of the mitochondria and an increase in permeability to facilitate the release of cytochrome C for the complex formation with caspase-9 and the subsequent activation of caspase-3 for apoptosis execution. This outcome also indicated that chlorophylls could be potential phytochemicals in inhibiting the growth of melanoma cells A375 through both the mitochondrial and death receptor pathways.

## 3. Materials and Methods

### 3.1. Materials

Pomelo leaves (*Citrus grandis* [Linn.] Osbeck) were obtained from a local farm located in Xinpu Town, Hsinchu County, Taiwan. Following freeze-drying, pomelo leaves were divided into several portions and placed into plastic bags separately for storage at −30 °C until use.

Both chlorophyll a and b standards, and internal standard Fast Green FCF were purchased from Sigma-Aldrich (St. Louis, MA, USA). The HPLC-grade solvents including methanol, ethanol, ethyl acetate, n-hexane, acetonitrile, dichloromethane, acetone and toluene were procured from Lab-Scan (Gliwice, Poland). The analytical-grade solvent n-hexane was from Grand Chemical (Taipei, Taiwan). Deionized water was prepared using a Millipore Milli-Q water purification system (Bedford, MA, USA). The adsorbents for preparative column chromatography, including magnesium oxide and diatomaceous earth, were, respectively, from Sigma-Aldrich and JT Baker (Phillipsburg, NJ, USA). Anhydrous sodium sulfate was from Nacalai Tesque (Kyoto, Japan). Tween 80 was from Yue Ba Enterprise Co., Ltd. (Taipei, Taiwan) while lecithin was from Gemfont Corporation (Taipei, Taiwan).

Human melanoma cells A375 (human malignant melanoma, BCRC 60031) were purchased from Bioresources Collection and Research Center (BCRC), Taiwan Food Industry Research and Development Institute (Hsinchu, Taiwan), while human fibroblast cells CCD986SK were provided by Professor Zhi-Feng Lu, Graduate Institute of Basic Medicine, Fu Jen Catholic University. Fetal bovine serum (FBS), Dulbecco’s modified Eagle’s medium (DMEM), the antibiotics penicillin-streptomycin and Hank’s balanced salt solution (HBSS) were from HyClone (Logan, UT, USA). Trypan blue stain (0.4%), 0.25% trypsin-EDTA and sodium pyruvate were from Gibco (Carlsbad, CA, USA). Propidium iodide (PI), RNase A, bovine serum albumin (BSA), sodium dodecyl sulfate (SDS), 3-(4.5-dimethylthoazol-2-yl)-2,5-diphenyltetrazolium bromide (MTT) and phosphoric acid were from Sigma-Aldrich. Sodium hydrogen carbonate was from FUJIFILM Wako Pure Chemical Corporation (Osaka, Japan). Bradford reagent for protein quantitation assay was from Bio-Rad Laboratories (Bossier City, LA, USA), while TEMED (N, N, N’, N’-tetramethylethylenediamine), ammonium persulfate (APS), glycine, Tris-base and 30% acrylamide were from USB Corporation (Cleveland, OH, USA). Amersham ECL prime western blotting detection reagent and PVDF blotting membrane were from GE Healthcare (Uppsala, Sweden). Caspase-8 and caspase-9 fluorometric assay kits were from BioVision (Milpitas, CA, USA), while caspase-3 fluorometric assay kit was from BD Biosciences (San Diego, CA, USA). 

For western blotting, the primary antibodies mouse monoclonal anti-β-actin was from Sigma-Aldrich, while rabbit anti-cyclin A and anti-Bax were from Millipore (Bedford, MA, USA). Rabbit anti-CDK2, anti-cytochrome C, anti-p21, anti-cyclin B and anti-Bcl-2 were from BD Biosciences (San Diego, CA, USA), while rabbit anti-CDC2 (CDK1) and rabbit anti-p53 were from Novus Biologicals (Littleton, CO, USA). The secondary antibodies peroxidase-conjugated goat anti-mouse IgG was from Jackson ImmunoResearch Lab (West Grove, PA, USA), while peroxidase-conjugated goat anti-rabbit IgG was from Anaspec (Fremont, CA, USA).

### 3.2. Instrumentation

The HPLC instrument (model 1100 series), composed of a G1316A thermostatted column compartment, a G1379A degasser, a G1311A quaternary pump, a G1312A binary pump and a G1315B photodiode array (PDA) detector was from Agilent Technologies (Santa Clara, CA, USA). The DP-4010 online degasser was from Sanwa Tsusho (Tokyo, Japan). The injector (Model 7161) was from Rheodyne (Rohnert Park, CA, USA). A 6130 Quadrupole mass spectrometer (MS) with a multimode ion source (ESI and APCI) was also from Agilent Technologies. An Eclipse XDB-C_18_ column (150 mm × 4.6 mm I.D., particle size 5 μm) used for chlorophyll separation was from Thermo Hypersil-Keystone (Bellefonte, PA, USA). The flow cytometer (model FC200) was from Beckman Coulter (Fullerton, CA, USA). The transmission electron microscope (TEM) (JEM-1400) was from JEOL (Tokyo, Japan). A Biospectrum 500 imaging system for luminescence/fluorescence imaging analysis was from UVP (Upland, CA, USA), while an electrophoresis system was from Bio-Rad Laboratories (Bossier city, LA, USA).

### 3.3. Pomelo Leave Extraction and Analysis

Extraction of chlorophylls in pomelo leaves was performed using a method described by Inbaraj et al. [33] and modified. A 10 g sample of Taiwan pomelo leave powder was added to 80 mL of n-hexane/ethanol/acetone/toluene (10:6:7:7, *v*/*v*/*v*/*v*) and left at room temperature for 1 h with shaking. Then, 80 mL of n-hexane was added and the mixture was shaken for 10 min, followed by adding 30 mL of 10% anhydrous sodium sulfate solution for partition. The upper layer containing both chlorophylls and carotenoids was collected. Extraction was continued by adding 30 mL of n-hexane to the bottom layer, and this procedure was repeated 4 times until the supernatant became colorless. Then, all the supernatants were combined and dried under reduced pressure, the residue was re-dissolved in 10 mL of n-hexane and the crude extract containing both chlorophylls and carotenoids was obtained. The crude extract was filtered through a 0.45 μm nylon membrane syringe filter and placed in an amber vial, followed by filling with nitrogen and storing at −80 °C for subsequent use.

### 3.4. Preparation of Chlorophylls from Pomelo Leave Extract by Preparative Column Chromatography

Isolation of chlorophylls from pomelo leave extract was conducted and modified based on a method in [34]. Initially, the adsorbent containing 10.5 g of magnesium oxide and diatomaceous earth (1:2.5, *w*/*w*) was loaded into a glass column (325 × 24 mm I.D.). Then, an appropriate amount of anhydrous sodium sulfate was added above the adsorbent with a height of about 1 cm. The column was then equilibrated with 20 mL of 100% n-hexane and 0.5 mL extract was added. Next, 25 mL of ethyl acetate/ethanol (98:2, *v*/*v*) was added to elute carotenoids, followed by adding 100 mL of acetone/ethanol (1:1, *v*/*v*) to elute chlorophylls. The chlorophyll eluate was collected and evaporated to dryness under reduced pressure, dissolved in 1 mL acetone/ethanol (1:1, *v*/*v*), passed through a 0.22 μm membrane filter, and 20 μL was injected into HPLC for qualitative and quantitative analyses.

### 3.5. HPLC Analysis of Chlorophylls

An HPLC method based on Loh, Inbaraj, Liu and Chen [34] was modified to separate and identify the various chlorophylls in pomelo leaves. An Eclipse XDB C18 column (150 × 4.6 mm I.D., 5 μm particle size) with a gradient mobile phase of acetone (A), acetonitrile (B) and methanol (C) was used: 2% A, 93% B and 5% C in the beginning, changed to 2% A, 71% B and 27% C in 0.3 min, 2% A, 64% B and 34% C in 6 min, 2% A, 45% B and 53% C in 9 min, 2% A, 39% B and 59% C in 21 min, 2% A, 24% B and 74% C in 24 min, 20% A, 0% B and 80% C in 26 min and returned to the original ratio in 30 min. The flow rate was 1 mL/min with detection wavelength at 660 nm and column temperature at 25 °C. The separation efficiency was evaluated based on retention factor (k). The identification was performed by comparing retention time, absorption spectra and mass spectra of unknown peaks with authentic standards. In addition, both absorption and mass spectra of chlorophylls reported in the literature was used for comparison. The mass spectra of various chlorophylls were determined with APCI mode using the following conditions: scanning range, 500–1000; drying gas flow, 5 L/min; nebulizer pressure, 20 psi; dry gas temperature, 350 °C; vaporizer temperature, 250 °C; capillary voltage, 2000 V; charging voltage, 2000 V; corona current, 4 μA and fragmentor voltage, 100 V. Additionally, the selected ion monitoring (SIM) mode was used to enhance sensitivity during MS detection. The peak purity of each chlorophyll was determined by a photodiode array detector automatically based on the Agilent G2180A Spectral Evaluation Software through measurement of degree in overlapping of absorption spectra within each peak or between unknown peaks and reference standards.

For quantitation, five concentrations, of 1, 5, 10, 20 and 50 μg/mL were each prepared for chlorophyll a and chlorophyll b, separately, in acetone, Additionally, five more concentrations of 100, 150, 200, 250 and 300 μg/mL were prepared for chlorophyll a. Each standard solution was then mixed with 20 μg/mL of Fast Green FCF (internal standard) each and injected into HPLC twice, and the standard calibration curves were prepared by plotting the concentration ratio (chlorophyll standard vs internal standard) against its area ratio, with the linear regression equation and coefficient of determination (R^2^) being obtained automatically based on an EXCEL software system. The various chlorophylls were quantified using a formula as described in a previous study [34].

### 3.6. Preparation of Chlorophyll Nanoemulsion

A sample of chlorophyll extract (3 mL) containing chlorophyll at 3.1 mg/mL was evaporated to dryness and then 0.1 g soybean oil (1%) and 0.018 g vitamin E (0.18%) was added in a test tube. The mixture was stirred thoroughly and then 0.8 g of Tween 80 (8%) and 0.1 g of lecithin (1%) was added. Again, the mixture was stirred thoroughly and 8.982 g of deionized water (89.82%) was added. The mixture was stirred thoroughly and sonicated at 4 °C for 1 h to obtain a clear and transparent chlorophyll nanoemulsion (10 mL).

### 3.7. Determination of Nanoemulsion Characteristics

A portion of chlorophyll nanoemulsion (30 μL) was diluted 10-fold with 25 mM potassium dihydrogen phosphate buffer solution (pH 5.3–5.5), passed through a 0.45 μm nylon membrane filter and placed in a polystyrene tube. The particle size distribution and polydispersity index (PDI) were determined by a dynamic light scattering (DLS) instrument using a BIC Particle Sizing 90 Plus software. In addition, a portion of chlorophyll nanoemulsion (10 μL) was diluted 120-fold with deionized water, and the zeta potential was measured by a zeta meter at 25 °C.

For TEM determination, a portion of chlorophyll nanoemulsion (10 μL) was diluted 100-fold with deionized water, and 20 μL was collected and added dropwise on the carbon-coated copper grid and allowed to settle for 30 s. A glass filter paper was used to remove excess sample, followed by adding 20 μL of 2% phosphotungstic acid (PTA) to negatively stain the background, and then, removal of excess PTA dye using a glass filter paper. Then, the sample was dried in an incubator overnight for TEM determination under 300,000× magnification using a voltage of 120 kVa. The shape and morphology of nanoemulsion were determined.

For determination of encapsulation efficiency, a sample of chlorophyll nanoemulsion (100 μL) was diluted 10-fold with 25 mM potassium dihydrogen phosphate buffer solution (pH 5.3–5.5). Then, the mixture was added to a membrane-containing dialysis tube with MW cut-off at 3 kDa and centrifuged at 12,000 rpm (25 °C) for 20 min. The nonencapsulated chlorophyll passed through the dialysis membrane into filtrate in the bottom layer, and the free chlorophyll concentration in the filtrate was analyzed by HPLC, after which the encapsulation efficiency (%) was calculated using a formula described in a previous study [16].

### 3.8. Cell Culture Study

Human malignant melanoma A375 cells were cultured in minimum essential medium (MEM) containing 10% FBS, 2 mM L-glutamine and Earle’s BSS, and supplemented with 1.5 g/L sodium bicarbonate, 0.1 mM nonessential amino acids, and 1.0 mM sodium pyruvate. Human pulmonary fibroblasts cells (CCD-986SK) were cultured in Dulbecco’s modified Eagle’s medium (DMEM) containing 10% FBS, while DMEM was supplemented with 4 mM L-glutamine and 4.5 g/L glucose.

Cells were collected from liquid nitrogen and placed immediately in a 37 °C water bath until completely thawed. The thawed cell solution containing 7% DMSO was added to a Petri dish, and 10 mL of MEM containing 10% FBS was added slowly. Then, cells were transferred to an incubator (37 °C and 5% CO_2_) and cultured until 80–90% confluency. After washing with PBS, 1 mL of 0.25% trypsin-EDTA was added for reaction, followed by adding medium (1 mL), centrifuging, removing the supernatant, adding medium (1 mL) and collecting a portion of cells in a new medium for cell number count. Then, cells were resuspended in culture medium, and 20 µL of the cell suspension was mixed with an equal amount of trypan blue and added to two chambers of a hemocytometer. The cell number in each chamber was then counted and converted to concentration [16].

### 3.9. MTT Assay

A375 cells were seeded in a 96-well plate at a density of 5 × 10^6^/well and incubated for 24 h. Following cell adherence to the plate, the culture medium was aspirated and cells washed once with PBS. Next, both chlorophyll extracts and nanoemulsions at a concentration of 2, 5, 10, 15 and 20 μg/mL were added to cells separately. Each concentration was assayed in triplicate. After 48-h incubation, the culture medium was aspirated, cells were washed with PBS, 200 μL of MTT solution (0.5 mg/mL) added to each well, and plates incubated for 1 h in the dark. Then the MTT solution was aspirated and 200 μL of DMSO added to dissolve purple crystals, and the absorbance at 570 nm was measured using an ELISA plate reader. The cell viability expressed as percentage was calculated using a formula described in a previous study [16].

### 3.10. Cell Cycle Analysis

A method similar to that used by Yang, Hung and Chen [16] was carried out for cell cycle analysis. In brief, cells (5 × 10^5^) were seeded in a 6-well plate and cultured for 24 h for cell adhesion, followed by removing the medium, washing with PBS and adding different concentrations of chlorophyll extracts or nanoemulsions (5 and 10 μg/mL) for further incubation for 48 h. Then, cells were washed with PBS, suspended with trypsin-EDTA, centrifuged to remove supernatant, washed again with PBS twice, added with 70% ethanol solution, stood overnight at 4 °C for cell fixing, centrifuged again to remove ethanol, washed with PBS and added with RNase (0.1 mL) and the staining agent PI for reaction at 37 °C in the dark for 30 min. Next, the stained cells were analyzed by a flow cytometer for cell cycle distribution after filtration.

### 3.11. Western Blotting

A method similar to that used by Yang, Hung and Chen [16] was used for Western blotting. In brief, cells (5 × 10^5^) were seeded in a 10-cm plate and cultured for 24 h, after which the medium was removed and various concentrations of chlorophyll extracts or nanoemulsions were added for further incubation for 48 h. After washing, cells with PBS and trypsin-EDTA for cell suspension, both medium and suspension were collected in a tube for centrifugation at 1000 rpm for 5 min, followed by removing the supernatant, washing cells with PBS, adding lysis buffer, centrifuging again at 12,000 rpm for 30 min and collecting the supernatant as protein extract. For protein quantitation, various concentrations (100, 200, 400, 500, 600, 800, 1000 μg/mL) of bovine serum albumin (BSA) standard containing Bradford coloring agent were prepared and the standard curve was obtained by plotting concentration against absorbance at 595 nm using an ELISA reader. Then, the linear regression equation was obtained for calculation of protein concentration. Next, the protein extract was collected and sample buffer added for SDS-PAGE electrophoresis in a tank with the upper and lower voltage being 60 V and 90 V, respectively, for protein separation. Then the PVDF membrane was activated in methanol, followed by soaking in transfer buffer, incubating, soaking in blocking buffer, adding TBST for washing 5 times and adding the primary antibody shown below: cytochrome C (1:1250), p21 (1:500), Bcl-2 (1:1250), p53 (1:500), cyclin B (1:500), cyclin A (1:500), CDK1 (1:500), CDK2 (1:500), Bax (1:500), β-actin (1:1250). After reacting at 4 °C overnight, the antibody was removed and washed with TBST 5 times. Then, the horseradish peroxidase (HPR) secondary antibody, anti-mouse IgG and anti-rabbit IgG were added and washed with TBST 5 times, followed by adding ECL for detection by a fluorescence image system and quantitation by a Gelpro 32 software system.

### 3.12. Activities of Caspase-3, Caspase-8 and Caspase-9

A fluorometric assay kit was used to determine activities of caspase-3, caspase-8 and caspase-9. Briefly, a sample of cell lysate (25 μL) was collected and mixed with 100 μL of 1X HEPES buffer containing 2.5 μL of substrate Ac-DEVD-AMC for reaction in a 37 °C water bath for 1 h, followed by transferring to a 96-well plate for absorbance measurement with excitation wavelength at 400 nm and emission wavelength at 505 nm by employing a multimode microplate reader for determination of caspase-3 activity. Likewise, for determination of both caspase-8 and caspase-9 activities, a sample of cell lysate (25 μL) was collected and mixed with 50 μL of 2× reaction buffer containing 5 μL of substrate IETD-AFC for caspase-8 or LEHD-AFC for caspase-9, for reaction in a 37 °C water bath for 1 h, followed by transferring to a 96-well plate for absorbance measurement with excitation wavelength at 400 nm and emission wavelength at 505 nm by using a multimode microplate reader.

### 3.13. Statistical Analysis

All the data were subjected to statistical analysis by using a software system [35]. In addition, the ANOVA analysis and Duncan’s multiple range test for significance in mean comparison (*p* < 0.05) was conducted.

## 4. Conclusions

An HPLC method for the separation and quantitation of chlorophylls a and b in pomelo leaves was established using an Eclipse XDB-C_18_ column and a gradient mobile phase of acetone (A), acetonitrile (B) and methanol (C), with flow rate at 1 mL/min and detection wavelength at 660 nm. An appropriate proportion of chlorophyll extract, lecithin, Tween 80 and deionized water was used for the preparation of chlorophyll nanoemulsion with an average particle size of 13.2 nm, zeta potential of −64.4 mV and encapsulation efficiency of 99%. Additionally, this nanoemulsion showed high thermal and storage ability. Chlorophyll nanoemulsion was more effective than chlorophyll extract in inhibiting the growth of human melanoma cells A375 through the elevation of expressions of cyclin A, cyclin B, p53 and p21, as well as the reduction of CDK1 and CDK2 expressions. In addition, both chlorophyll extract and nanoemulsion could increase the expressions of Bax and cytochrome C, while decreasing Bcl-2 expression for the activation of caspase-3, caspase-8 and caspase-9 for subsequent cell apoptosis. Further research is necessary to study the inhibition mechanism of skin tumors in vivo as affected by chlorophyll extract and nanoemulsion.

## Figures and Tables

**Table 1:** Retention time (t_R_), retention factor (k), separation factor (α) and contents (µg/g) of chlorophylls in Pomelo leaves.

Peak No	Compound	t_R_ (min)	Retention Factor (k) ^a^	Content (µg/g) ^b^	*m*/*z* Found	*m*/*z* Reported	λ_max_ (On-Line)	λ_max_ (Reported)
1	Chlorophyll b	13.92	3.35	785.8	907.4 [M + H] 630.4 [M + H−278]	907.4 [M + H] 629.4 [M + H−278] ^c^	464, 600, 648	464, 599, 648 ^c^
2	Chlorophyll a	22.54	6.04	2278.3	894.6 [M + H]	893.6 [M + H] ^c^	430, 618, 664	431, 617, 663 ^c^

^a^ k = (t_R_ − t_0_)/t_0_, where t_R_ is the retention time of peak 1 or peak 2, while t_0_ is the retention time of solvent peak. ^b^ Average of duplicate analyses. ^c^ Based on a report by Delpino-Rius, Cosovanu, Eras, Vilaró, Balcells and Canela-Garayoa [17].

**Table 2:** Particle size, polydispersity index (PDI), zeta potential and pH of chlorophyll nanoemulsion during storage at 4 °C for 90 days.

	Average Particle Size ^a^	PDI ^a^	Zeta Potential ^a^	pH ^a^
0	13.2 ± 0.10 ^A^	0.15 ± 0.01 ^A^	−64.4 ± 1.10 ^A^	4.85 ± 0.08 ^A^
7	13.5 ± 0.15 ^A^	0.183 ± 0.01 ^AB^	−66.1 ± 0.82 ^B^	4.80 ± 0.13 ^AB^
14	14.6 ± 0.38 ^B^	0.193 ± 0.01 ^AB^	−66.2 ± 1.36 ^B^	4.77 ± 0.14 ^AB^
21	15.0 ± 0.40 ^B^	0.21 ± 0.01 ^BC^	−66.1 ± 0.75 ^B^	4.75 ± 0.11 ^AB^
28	15.7 ± 0.44 ^CD^	0.235 ± 0.04 ^BC^	−67.3 ± 0.21 ^BC^	4.58 ± 0.17 ^BC^
35	15.7 ± 0.44 ^CD^	0.268 ± 0.03 ^BC^	−68.6 ± 0.26 ^C^	4.52 ± 0.35 ^BC^
42	16.0 ± 0.93 ^CDE^	0.278 ± 0.04 ^BC^	−70.4 ± 0.61 ^D^	4.50 ± 0.34 ^CD^
60	16.4 ± 0.26 ^DE^	0.289 ± 0.04 ^BC^	−73.3 ± 1.20 ^D^	4.44 ± 0.12 ^CD^
90	16.6 ± 0.46 ^E^	0.295 ± 0.07 ^C^	−75.1 ± 0.51 ^D^	4.40 ± 0.07 ^D^

^a^ Data shown are mean ± standard deviation (*n* = 3). Data with different capital letters (A–E) in the same column are significantly different at *p* < 0.05.

**Table 3:** Particle size and zeta potential change as affected by chlorophyll nanoemulsion during heating at 40–100 °C for varied length of time.

Temp. (°C)	Particle Size (nm)					Zeta Potential (mV)				
	Heating Time					Heating Time				
	0 h	0.5 h	1 h	1.5 h	2 h	0 h	0.5 h	1 h	1.5 h	2 h
40	13.2	13.5	15.5	16.5	16.4	−64.6	−75.4	−73.3	−72.9	−67.5
60	13.2	13.8	13.7	12.6	12.4	−64.6	−64.4	−68.1	−64.1	−61.8
80	13.2	14.6	13.7	13.4	13.2	−64.6	−34.4	−33.3	−32.0	−28.6
100	13.2	14.3	14.2	13.7	12.6	−64.6	−44.6	−37.4	−35.5	−25.8

## Supplementary Materials

The following are available online at https://www.mdpi.com/article/10.3390/plants10081664/s1, Figure S1: Effect of chlorophyll extracts and nanoemulsions on cell cycle distribution of melanoma cells A375. Control represents cells incubated in medium only.
