# Supplementary material for: Preparation of Chlorophyll Nanoemulsion from Pomelo Leaves and Its Inhibition Effect on Melanoma Cells A375
Source: Plants (Basel). 2021 Aug 12;10(8):1664. doi: 10.3390/plants10081664 (PMC8398141; doi:10.3390/plants10081664)
Supplement: Supplementary file 1 [file plants-10-01664-s001.zip › plants-1334283-supplementary.pdf]

Supplementary information

For

Preparation of Chlorophyll Nanoemulsion from Pomelo Leaves and its Inhibition Effect on Melanoma Cells A375

Man-Hai Liu[1], Yi-Fen Li[2] and B. H. Chen[2,3,*]

[1] Department of Food Science, China University of Science and Technology, Taipei 11581, Taiwan.

[2] Department of Food Science, Fu Jen Catholic University, New Taipei City 24205, Taiwan.

[3] Department of Nutrition, China Medical University, Taichung 404, Taiwan.

* Correspondence: 002622@mail.fju.edu.tw; Tel.: +886 2 2905 3626; Fax +886 2 2209 3271.

only.

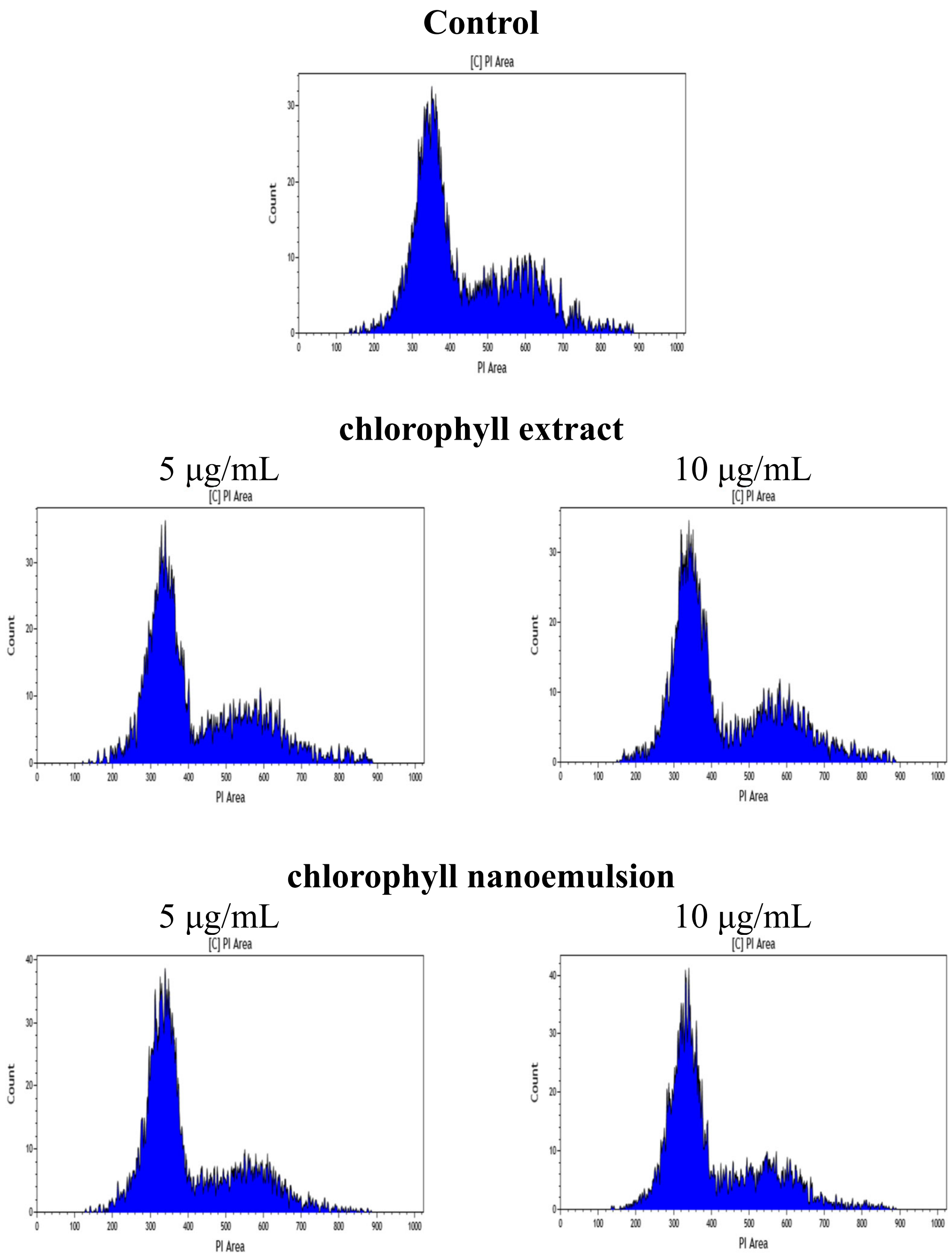

Figure S1. Effect of chlorophyll extracts and nanoemulsions on cell cycle distribution of melanoma cells A375. Control represents cells incubated in medium only.
